# Supplementary material for: CircPRMT5 promotes progression of osteosarcoma by recruiting CNBP to regulate the translation and stability of CDK6 mRNA
Source: PLoS One. 2024 Apr 16;19(4):e0298947. doi: 10.1371/journal.pone.0298947 (PMC11020494; doi:10.1371/journal.pone.0298947)
Supplement: S1 Table — (DOCX) [file pone.0298947.s001.docx]

**S1 Table**: Primers sequences for qRT-PCR.

|  | Forward (5’-3’) | | Reverse (5’-3’) | |
| --- | --- | --- | --- | --- |
| CircPRMT5  (Divergent) | TCATGTCATTGATCGCTGGC | | CTGTGTGTGTAGTTGGTGCA | |
| CircPRMT5  (Convergent) | ACTTCCGGACTTTGTGTGAC | | ATGAGCCTCTGGTGCATCTT | |
| PRMT5 | CTGTCTTCCATCCGCGTTTCA | | GCAGTAGGTCTGATCGTGTCTG | |
| GAPDH | TGCACCACCAACTGCTTAGC | | GGCATGGACTGTGGTCATGAG | |
| FRAS1 | CTAGCGTTGGCGGAATTTGC | | GCATTGGTTGGCAGCTATTTGA | |
| CDK6 | GCTGACCAGCAGTACGAATG | | GCACACATCAAACAACCTGACC | |
| ROBO2 | | CACCCAGAACCCACCATCTAC | | CACCTGGTTAATTGGCCTCCT |
