# Supplementary material for: CircPRMT5 promotes progression of osteosarcoma by recruiting CNBP to regulate the translation and stability of CDK6 mRNA
Source: PLoS One. 2024 Apr 16;19(4):e0298947. doi: 10.1371/journal.pone.0298947 (PMC11020494; doi:10.1371/journal.pone.0298947)
Supplement: S2 Table — (DOCX) [file pone.0298947.s002.docx]

**S2 Table**: Sequences information for gene knockdown.

|  | 5'-3' |
| --- | --- |
| sh-circPRMT5-1 | CCGGGGCTCCTCAAGTTCTGGATGCCTCGAGGCATCCAGAACTTGAGGAGCCTTTTTG (sense)  AATTCAAAAAGGCTCCTCAAGTTCTGGATGCCTCGAGGCATCCAGAACTTGAGGAGCC (antisense) |
| sh-circPRMT5-2 | CCGGCTCAAGTTCTGGATGCGGGTACTCGAGTACCCGCATCCAGAACTTGAGTTTTTG (sense)  AATTCAAAAACTCAAGTTCTGGATGCGGGTACTCGAGTACCCGCATCCAGAACTTGAG (antisense) |
| sh-CDK6-1 | CCGGGATCAAGACTTGACCACTTACCTCGAGGTAAGTGGTCAAGTCTTGATCTTTTTG (sense)  AATTCAAAAAGATCAAGACTTGACCACTTACCTCGAGGTAAGTGGTCAAGTCTTGATC (antisense) |
| sh-CDK6-2 | CCGGGGATATGATGTTTCAGCTTCTCTCGAGAGAAGCTGAAACATCATATCCTTTTTG (sense)  AATTCAAAAAGGATATGATGTTTCAGCTTCTCTCGAGAGAAGCTGAAACATCATATCC (antisense) |
