# Supplementary figures and images for: CircPRMT5 promotes progression of osteosarcoma by recruiting CNBP to regulate the translation and stability of CDK6 mRNA
Source: PLoS One. 2024 Apr 16;19(4):e0298947. doi: 10.1371/journal.pone.0298947 (PMC11020494; doi:10.1371/journal.pone.0298947)

Fig. 3

3C

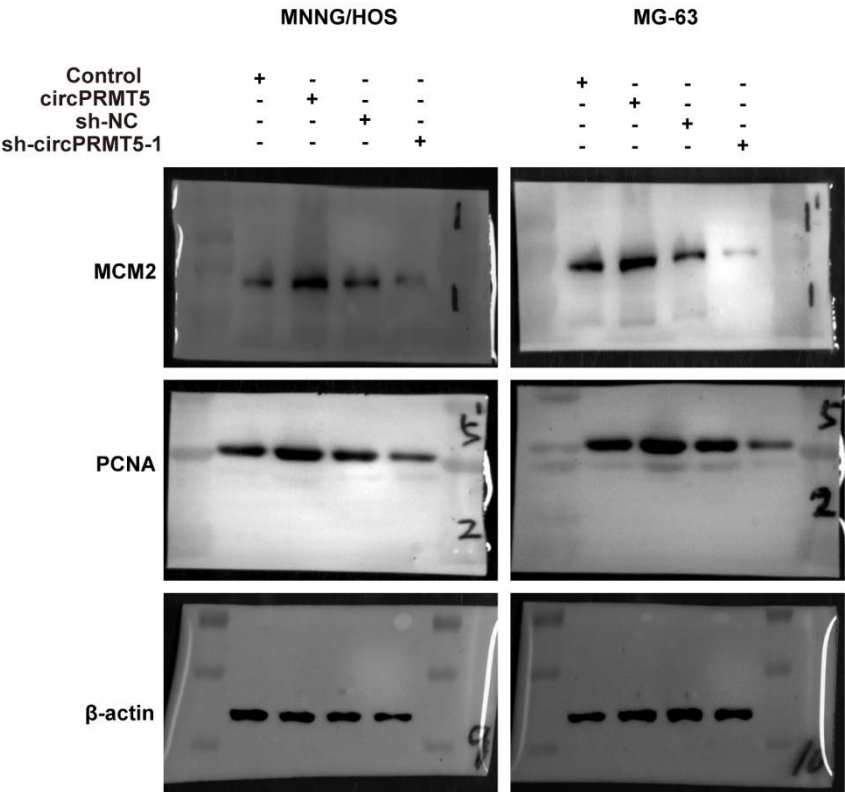

Fig. 4

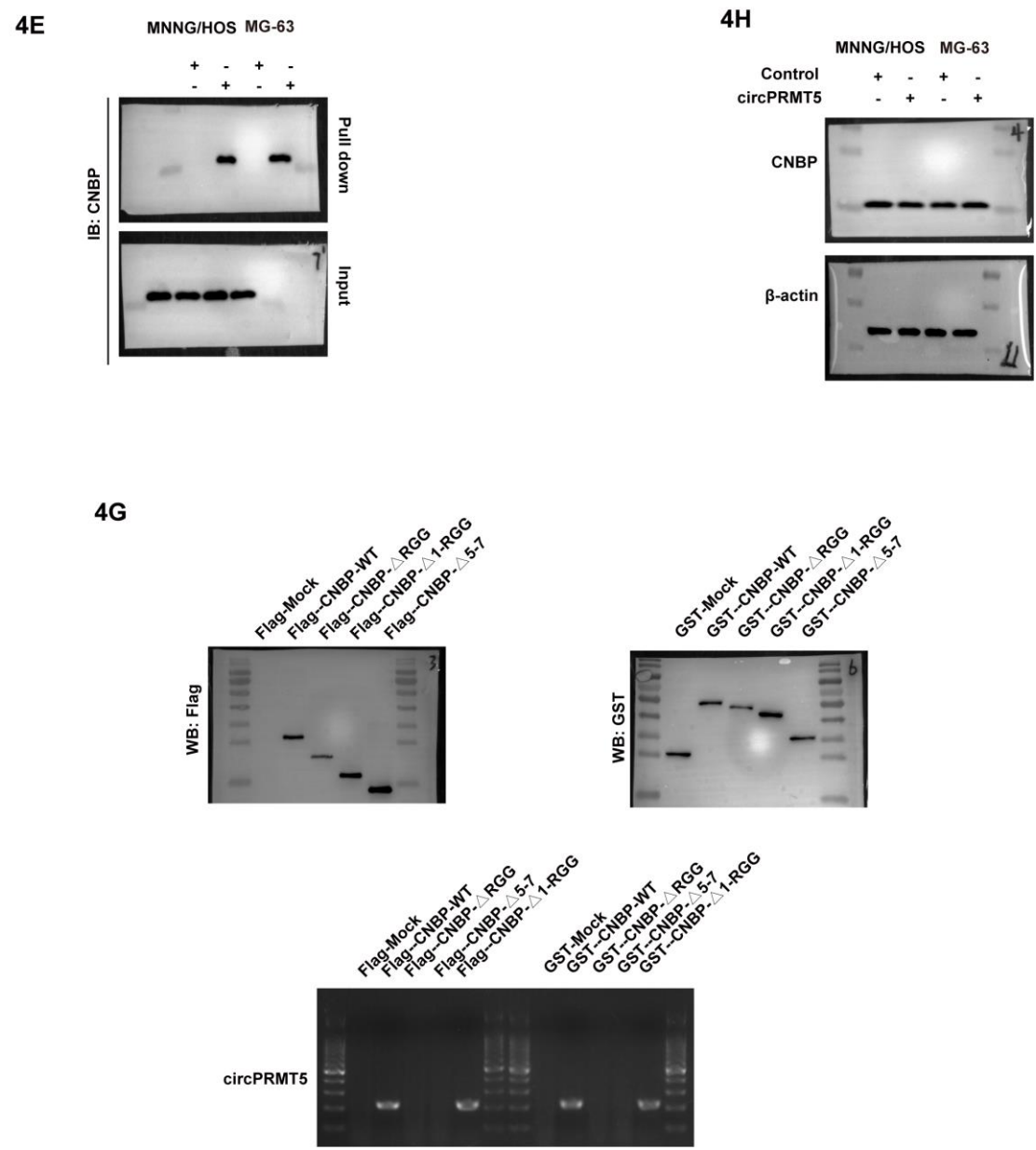

Fig. 5

5H

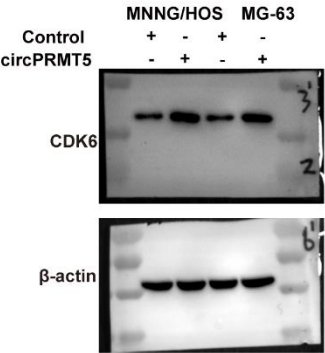

5I

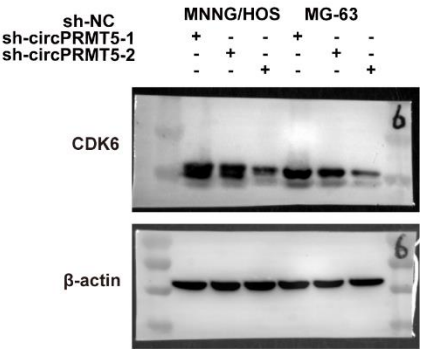

Fig. 6

6A

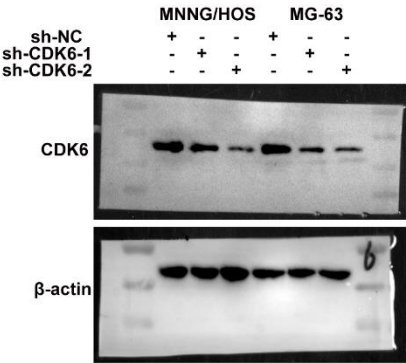

Supplement: S1 Raw images — (PDF) [file pone.0298947.s004.pdf]
