# Supplementary material for: CircPRMT5 promotes progression of osteosarcoma by recruiting CNBP to regulate the translation and stability of CDK6 mRNA
Source: PLoS One. 2024 Apr 16;19(4):e0298947. doi: 10.1371/journal.pone.0298947 (PMC11020494; doi:10.1371/journal.pone.0298947)
Supplement: S1 Appendix — (DOCX) [file pone.0298947.s005.docx]

**Supplementary Table S1**: Primers sequences for qRT-PCR.

|  | Forward (5’-3’) | Reverse (5’-3’) |
| --- | --- | --- |
| circPRMT5  (Divergent) | TCATGTCATTGATCGCTGGC | CTGTGTGTGTAGTTGGTGCA |
| circPRMT5  (Convergent) | ACTTCCGGACTTTGTGTGAC | ATGAGCCTCTGGTGCATCTT |
| PRMT5 | CTGTCTTCCATCCGCGTTTCA | GCAGTAGGTCTGATCGTGTCTG |
| GAPDH | TGCACCACCAACTGCTTAGC | GGCATGGACTGTGGTCATGAG |
| FRAS1 | CTAGCGTTGGCGGAATTTGC | GCATTGGTTGGCAGCTATTTGA |
| CDK6 | GCTGACCAGCAGTACGAATG | GCACACATCAAACAACCTGACC |
| ROBO2 | CACCCAGAACCCACCATCTAC | CACCTGGTTAATTGGCCTCCT |

**Supplementary Table S2**: sequences information for gene knockdown.

|  | 5'-3' |
| --- | --- |
| sh-circPRMT5-1 | CCGGGGCTCCTCAAGTTCTGGATGCCTCGAGGCATCCAGAACTTGAGGAGCCTTTTTG (sense)  AATTCAAAAAGGCTCCTCAAGTTCTGGATGCCTCGAGGCATCCAGAACTTGAGGAGCC (antisense) |
| sh-circPRMT5-2 | CCGGCTCAAGTTCTGGATGCGGGTACTCGAGTACCCGCATCCAGAACTTGAGTTTTTG (sense)  AATTCAAAAACTCAAGTTCTGGATGCGGGTACTCGAGTACCCGCATCCAGAACTTGAG (antisense) |
| sh-CDK6-1 | CCGGGATCAAGACTTGACCACTTACCTCGAGGTAAGTGGTCAAGTCTTGATCTTTTTG (sense)  AATTCAAAAAGATCAAGACTTGACCACTTACCTCGAGGTAAGTGGTCAAGTCTTGATC (antisense) |
| sh-CDK6-2 | CCGGGGATATGATGTTTCAGCTTCTCTCGAGAGAAGCTGAAACATCATATCCTTTTTG (sense)  AATTCAAAAAGGATATGATGTTTCAGCTTCTCTCGAGAGAAGCTGAAACATCATATCC (antisense) |

**Supplementary Table S3**: antibody information used in this study.

| Antibody | Application | Cat number | Company |
| --- | --- | --- | --- |
| MCM2 | WB | 10513-1-AP | Proteintech |
| PCNA | WB | 10205-2-Ap | Proteintech |
| β-actin | WB | 81115-1-RR | Proteintech |
| AGO2 | IP | 67934-1-Ig | Proteintech |
| UPF2 | IP | A303-929A-T | ThermoFisher |
| SFPQ | IP | 15585-1-AP | Proteintech |
| TIAL1 | IP | ab129499 | abcam |
| FUS | IP | 11570-1-AP | Proteintech |
| CNBP | WB/IP | 67109-1-Ig | Proteintech |
| Flag | WB | 66008-4-Ig | Proteintech |
| GST | WB | 10000-0-AP | Proteintech |
| CDK6 | WB | 14052-1-AP | Proteintech |
